# Usefulness of Adalimumab in the Treatment of Refractory Uveitis Associated with Juvenile Idiopathic Arthritis

**DOI:** 10.1155/2013/560632

**Published:** 2013-12-30

**Authors:** Carmen García-De-Vicuña, Manuel Díaz-Llopis, David Salom, Rosa Bou, Jesus Díaz-Cascajosa, Miguel Cordero-Coma, Gabriela Ortega, Norberto Ortego-Centeno, Marta Suarez-De-Figueroa, Juan Cruz-Martínez, Alex Fonollosa, Ricardo Blanco, Ángel María García-Aparicio, Jose M. Benítez-Del-Castillo, Jordi Antón

**Affiliations:** ^1^Ophthalmology Department, Hospital Sant Joan de Déu, Universidad de Barcelona, Passeig Sant Joan de Déu 2, Esplugues de Llobregat, 08950 Barcelona, Spain; ^2^Service of Ophthalmology, Hospital Universitari i Politècnic La Fe, and Facultad de Medicina, Universidad de Valencia, Valencia, Spain; ^3^Unit of Pediatric Rheumatology, Pediatrics Department, Hospital Sant Joan de Déu, Universidad de Barcelona, Esplugues del Llobregat, Barcelona, Spain; ^4^Service of Ophthalmology, Hospital de León, León, Spain; ^5^Laboratory of Ocular Immunology and Uveitis, Instituto Nacional de Rehabilitación, Calzada Mexico-Xochimilco, Mexico, DF, Mexico; ^6^Service of Internal Medicine, Hospital San Cecilio, Granada, Spain; ^7^Service of Ophthalmology, Hospital Ramón y Cajal, Madrid, Spain; ^8^Service of Rheumatology, Hospital de Pontevedra, Pontevedra, Spain; ^9^Service of Ophthalmology, Hospital de Cruces, Bilbao, Spain; ^10^Service of Rheumatology, Hospital Universitario Marqués de Valdecilla, Santander, Spain; ^11^Service of Rheumatology, Hospital Virgen de la Salud, Toledo, Spain; ^12^Service of Ophthalmology, Hospital Clínico San Carlos, Madrid, Spain

## Abstract

*Purpose*. To assess the efficacy and safety of adalimumab in patients with juvenile idiopathic arthritis (JIA) and associated refractory uveitis. *Design*. Multicenter, prospective case series. *Methods*. Thirty-nine patients (mean [SD] age of 11.5 [7.9] years) with JIA-associated uveitis who were either not responsive to standard immunosuppressive therapy or intolerant to it were enrolled. Patients aged 13–17 years were treated with 40 mg of adalimumab every other week for 6 months and those aged 4–12 years received 24 mg/m^2^ body surface. *Results*. Inflammation of the anterior chamber (2.02 [1.16] versus 0.42 [0.62]) and of the posterior segment (2.38 [2.97] versus 0.35 [0.71] decreased significantly between baseline and the final visit (*P* < 0.001). The mean (SD) macular thickness at baseline was 304.54 (125.03) **μ** and at the end of follow-up was 230.87 (31.12) **μ** (*P* < 0.014). Baseline immunosuppression load was 8.10 (3.99) as compared with 5.08 (3.76) at the final visit (*P* < 0.001). The mean dose of corticosteroids also decreased from 0.25 (0.43) to 0 (0.02) mg (*P* < 0.001). No significant side effects requiring discontinuation of therapy were observed. *Conclusion*. Adalimumab seems to be an effective and safe treatment for JIA-associated refractory uveitis and may reduce steroid requirement.

## 1. Introduction

Uveitis is a well-known extra-articular manifestation of spondyloarthritides, which may lead to severe functional impairment [1]. Childhood uveitis is relatively rare and may be secondary to a variety of causes. The majority of children with uveitis have idiopathic uveitis, with uveitis secondary to juvenile idiopathic arthritis (JIA) being the second most common diagnosis [2, 3]. Uveitis occurs in around 10–15% of the patients with JIA, although most reports are retrospective and describe referral centers' experiences rather than population-based studies [4]. It has been largely recognized that uveitis is a serious manifestation of JIA. Complications increase with the duration of the disease, with a potential for cataract, glaucoma, band keratopathy, synechiae, macular edema, and significant ocular damage with impaired vision and even blindness [5, 6]. Antinuclear antibody (ANA)—positive girls younger than 7 years of age with oligoarticular JIA are at the greatest risk of developing eye disease [7]. Presence of complications at first visit and uveitis manifestation before arthritis have been identified as predictors for complications [8, 9].

JIA-associated refractory chronic uveitis is a challenge for treatment. Topical and systemic corticosteroids are the first-line standard therapy, often reinforced by conventional disease-modifying antirheumatic drugs (DMARDs) including a variety of immunomodulatory agents, but methotrexate remains the most commonly used drug [10–12]. During the last few years, tumor necrosis factor-alpha (TNF-*α*) blocking agents (infliximab, etanercept, and adalimumab) have been used to treat chronic refractory uveitis in childhood [12–15] and, particularly, uveitis associated with JIA in children who have failed topical and second-line DMARD therapy [16, 17]. Adalimumab, a TNF-*α* antagonist, has shown remarkable efficacy in rheumatoid arthritis, ankylosing spondylitis, psoriatic arthritis, Crohn's disease, and plaque-type psoriasis. Currently, there is a large experience of the beneficial effects of adalimumab in the treatment of these conditions in daily practice. Since 2008, adalimumab has been approved for the treatment of active juvenile idiopathic arthritis (JIA).

Adalimumab has shown promising results in controlling intraocular inflammation, even though this has been used primarily as a rescue therapy for refractory uveitis [18]. Open-label evaluations have demonstrated the efficacy of adalimumab therapy for childhood uveitis [19, 20]. In a recent comparative cohort study on anti-TNF-*α* treatment for sight-threatening childhood uveitis, adalimumab was more efficacious than infliximab in maintaining remission of chronic childhood uveitis for over 3 years [21]. In recent data of a large retrospective cohort of children with JIA and refractory chronic uveitis, treatment with adalimumab for a mean of 2 years was associated with an overall improvement of disease activity in 57% of the cases [22]. In recent evidence-based interdisciplinary guidelines for anti-inflammatory treatment of uveitis associated with JIA, adalimumab is recommended as the preferred TNF-*α* inhibitor [23].

A prospective multicenter study was design to assess the efficacy and safety of adalimumab therapy in a cohort of patients with JIA and associated uveitis who were treated with adalimumab in daily practice.

## 2. Materials and Methods

A prospective open-label, noncomparative, and multicenter study was conducted in the outpatient clinics of the services of ophthalmology or uveitis units of 10 centers throughout Spain and 1 center in Latin America (Mexico, DF) in daily practice conditions. The objective of the study was to assess the efficacy and tolerability of adalimumab for treating children and adolescents with uveitis in conjunction with JIA. The diagnosis of JIA was based on the 2001 revised International League Against Rheumatism (ILAR) classification criteria [24]. All diagnoses of JIA were confirmed by a pediatric rheumatologist. To be considered eligible for this study, patients were required to have disease onset prior to 16 years, bilateral or unilateral, chronic, and noninfectious uveitis that was refractory to standard immunosuppressive therapy for uveitis, with systemic corticosteroids and at least one other immunosuppressive medication, or to be intolerant to such therapy. Refractory was considered as persistent active uveitis for at least 3 months despite systemic steroids and immunosuppressive treatment. Uveitis was classified anatomically according to the International Uveitis Study Group (IUSG) classification [25]. The study protocol was approved by the ethics committee of each participating center, and written informed consent was obtained from the parents of legal guardians as well as from the patients older than 16 years of age.

All patients underwent a protein-purified derivative (PPD) skin test and chest radiography before enrollment because of the risk of tuberculosis reactivation associated with TNF-*α* inhibition. Patients diagnosed with latent tuberculosis, defined as a PPD skin conversion consisting of an induration of 5 mm or larger without radiographic or clinical evidence of disseminated or pulmonary disease, received antituberculosis prophylaxis at least 3 weeks prior to the first dose of adalimumab.

In all patients, treatment with adalimumab was initiated because of active refractory uveitis. Children and adolescents between 13 and 17 years of age were treated with 40 mg of adalimumab (Humira, Abbott Laboratories, Madrid, Spain), subcutaneously (s.c.) every other week for 6 months. For children aged between 4 and 12 years, doses were administered as indicated in the product label (i.e., 24 mg/m^2^ body surface area up to a maximum single dose of 40 mg s.c. every other week). The volume of injection is selected according to the weight and height of the patient. Patients (or parents in case of young children) were instructed by a specialized nurse in self-administration of adalimumab.

Outcome variables included intraocular inflammation, visual acuity, immunosuppression load, and macular thickness. Inflammatory activity was graded according to the SUN Working Group grading schemes [26] for the anterior (cells and flare) and posterior (vitreous cells and haze) chambers, from grade 0 to 4. Worsening was defined as a two-step increase in the level of inflammation or as an increase from 3+ to 4+ (this was also the adopted definition for relapse during follow-up). Improvement was defined as a two-step decrease in the level of inflammation or decrease to grade 0.

The best-corrected visual acuity (BCVA) was measured according to the ETDRS protocol adapted by the Age Related Eye Disease Study [27]. Three lines (±0.3 logMAR; ±15 letters) of change were chosen as the standard for worsening or improvement in visual acuity, because as reported in previous uveitis clinical trials, this was the minimum necessary number to reflect a clinical significant change.

The immunosuppression load was assessed with a semiquantitative scale for each medication as described by Nussenblatt et al. [28]. The grading scheme provides a combined, single numeric score for the total immunosuppression load per unit of body weight per day. Grades for each agent (prednisone, cyclosporine, azathioprine, methotrexate, and chlorambucil) ranged on a scale from 0 to 9. For patients receiving multiple medications, the sum of the grading scores for each drug was used to calculate the total immunosuppression score at baseline visit and at the end of follow-up on a scale from 0 to 15. Topical or periocular corticosteroid therapy was excluded from the calculation of the immunosuppression load. A ≥50% reduction in the immunosuppression load was considered a significant reduction in the associated immunosuppressant therapy.

Macular thickness was measured by optical coherence tomography (OCT) (Stratus OTC, Carl Zeiss Meditec, Dublin, CA, USA). Changes in the mean 1 mm central foveal retinal thickness served to evaluate the reduction in the cystoid macular edema (CME).

*Statistical Analysis*. Categorical data are expressed as absolute number and percentages and continuous data as mean and standard deviation (SD). Continuous variables were analyzed with the paired Student's *t*-test (e.g., immunosuppression load and macular thickness) and qualitative variables (degree of intraocular inflammation) with the Wilcoxon signed-rank test. Statistical significance was set at *P* < 0.05. The analysis was performed using the Statistical Package for the Social Sciences (SPSS) version 12.0 for Windows.

## 3. Results

A total of 39 patients, 11 boys and 28 girls, with a mean (SD) age of 11.5 (7.9) years were included in the study. All patients presented initially with arthritis. The age at diagnosis of arthritis ranged between 18 months and 6 years, with a median of 4.4 years. Manifestations of uveitis developed approximately 8 months after the diagnosis of JIA.

All patients were followed for at least 6 months. Twenty-nine (74.4%) patients had chronic anterior uveitis, 1 (2.6%) intermediate and anterior uveitis and 9 (23.1%) panuveitis (anterior with secondary posterior uveitis). The right eye was involved in 2 (5.1%) patients, the left eye in 5 (12.8%), and both eyes in the remaining 32 (82.1%) patients.

Changes of outcome variables associated with adalimumab therapy are shown in Table 1. A marked decrease of intraocular inflammation with adalimumab at the end of follow-up was observed. The baseline anterior chamber degree of inflammation was 2.02 (1.16) and the posterior chamber degree of inflammation was 2.38 (2.97) on the standardized scale, and at the end of follow-up they were 0.42 (0.62) and 0.35 (0.71), respectively. There was a statistically significant difference in the mean degree of intraocular inflammation of the anterior chamber between the initial visit and at the end of follow-up (*P* < 0.001). Differences in the mean degree of intraocular inflammation of the posterior chamber were also statistically significant (*P* < 0.005).

The visual acuity improved by −0.3 logMAR (+15 letters) in 4 (12.5%) eyes out of 32 eyes, remained stable in 26 (81.2%) eyes, and worsened by +0.3 logMAR (−15 letters) in 2 (6.25%) eyes at the end of follow-up. The baseline visual acuity was +0.30 ± 0.32 (mean ± SD) logMAR and +0.24 ± 0.35 logMAR at the end of follow-up. There was no statistically significant difference between the mean baseline visual acuity and the mean visual acuity at the end of follow-up (*P* = 0.226).

**Figure 1:**
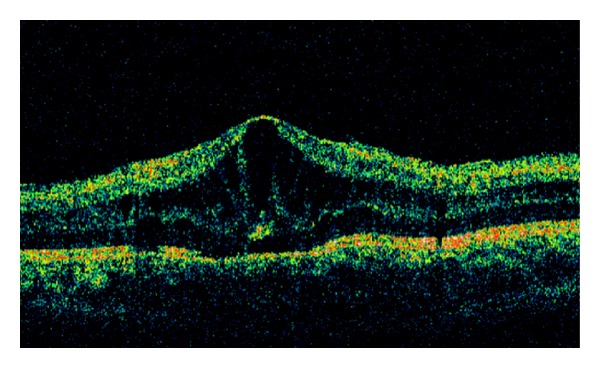
Ocular coherence tomography in a patient with JIA and cystoid macular edema, with a macular thickness of 431 **μ** at baseline.

**Figure 2:**
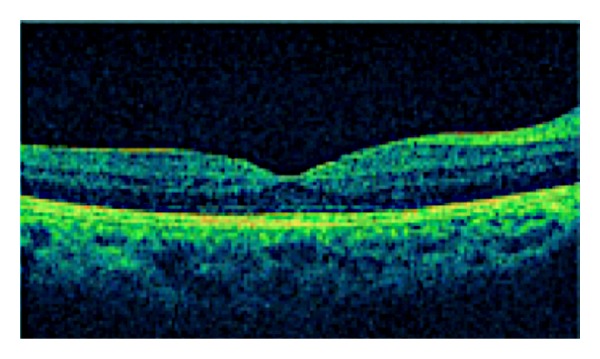
Complete resolution with macular thickness of 278 *μ* after treatment with adalimumab.

The OCT macular examination revealed that 3 eyes (7.7%) had CME at baseline, and at the end of follow-up, there was complete resolution of CME in all cases (Figure 1). The mean (SD) macular thickness at baseline was 304.54 (125.03) *μ* and at the end of follow-up was 230.87 (31.12) *μ*. There was a statistically significant difference between pretreatment and final macular thickness (*P* < 0.014) (Figure 2).

Baseline immunosuppression load was 8.10 (3.99) as compared with 5.08 (3.76) at the final visit. There was a significant difference in the mean immunosuppression load between baseline and end of follow-up (*P* < 0.001).

Treatment with adalimumab was also associated with a decrease in the mean dose of corticosteroids from 0.25 (0.43) at the pretreatment visit to 0 (0.0.2) mg at the end of follow-up (*P* < 0.001).

Adalimumab was well tolerated in all patients during the 6-month follow-up period except for the case of a 9-year-old girl that at the fifth month of administration of adalimumab had a severe uveitis reactivation that needed a switch to infliximab therapy to control the episode. Minor side effects at the site of injection, such as pain, erythema, localized rash, or minor hemorrhage were the most common side effects observed. During the follow-up period, 3 (7.8%) patients experienced reactivation of uveitis, which was severe in only 1 patient (3.1%), who discontinued the adalimumab therapy and improved with infliximab treatment. The remaining 2 patients did not discontinue the adalimumab therapy because the inflammation was controlled with periocular steroid injection of the affected eye.

## 4. Discussion

This prospective study carried out in a clinical series of 39 children and adolescents with JIA-associated uveitis confirms the value of adalimumab as a treatment option in patients who were poorly responsive to conventional therapy for uveitis. The effectiveness of adalimumab was demonstrated by a statistically significant decrease of anterior chamber and vitreous cavity degree of inflammation, reduction of macular thickness, and decrease of the immunosuppression load.

Most reported treatment studies in pediatric uveitis are retrospective case series, which may be explained by the relative rarity of uveitis associated with JIA, urgency of treatment to prevent complications, difficulty in examining younger children, and the lack of controlled clinical trials [4]. The course of uveitis is chronic with waxing and waning activity, and follow-up times should be longer enough to assess the efficacy of treatment [4]. TNF-*α* appears to play a role in the pathogenesis of uveitis [29–31] and therefore TNF-*α* blockade is a rational therapy for uveitis refractory to standard treatment [16].

Adalimumab is currently considered the most efficacious TNF-*α* blocker for childhood uveitis and the preferred biologic drug for the treatment of uveitis associated with JIA [23]. In this respect, data of the present study collected in a prospective series of patients with JIA and refractory uveitis add evidence to the preferential role of adalimumab in the therapeutic armamentarium of this condition. The beneficial effects of adalimumab in reducing ocular inflammation together with the good tolerability profile of this agent are consistent with data previously published in the literature. Previous studies, however, are generally retrospective or small series of case studies, including 8 patients reported by Sen et al. [32], 6 patients described by Katsicas and Russo [33], 3 patients reported by Foeldvari et al. [12] among 47 patients with JIA-related uveitis treated with anti-TNF-*α* collected in a multinational survey, and 9 patients reported by Vazquez-Cobian et al. [17]. Other clinical studies with a larger number of patients include a group of 17 patients reported by Biester et al. [20] and a retrospective observational study of 20 patients with JIA and chronic uveitis reported by Tynjälä et al. [34]. Findings of these studies in children with JIA who were poorly responsive to conventional therapy for uveitis are consistent with a decrease in ocular inflammation, sustained response, and decrease or discontinuation of other immunosuppressive agents. In a recent retrospective cohort of 54 patients with JIA treated with adalimumab for active associated uveitis, improvement of ocular inflammation was recorded in 28% of the cases [21]. In this study, in which treatment with adalimumab was given for a mean of 2 years, at the end of the follow-up, only 4 patients remained on adalimumab monotherapy and the rest were on combined therapy with prednisone, methotrexate, cyclosporine A, or other antimetabolite drugs.

The three TNF-*α* antagonists (etanercept, infliximab, and adalimumab) appear to have similar efficacy in rheumatoid arthritis (JIA), but this does not appear to be the case with uveitis where infliximab seems to be more effective than etanercept, and adalimumab more effective than infliximab. In an open-label prospective comparison of infliximab and adalimumab in 33 children with refractory noninfectious uveitis, 22 of them with associated JIA, at 40 months of follow-up, 60% of children on adalimumab compared to 18.8% of children on infliximab were still in remission on therapy [21].

In summary, treatment with adalimumab for at least 6 months was associated with improvement of symptoms and decrease in inflammatory activity in 39 patients with refractory uveitis associated with JIA. It also allowed a significant reduction of concomitant immunosuppressive therapies.

## Figures and Tables

**Table 1:** Comparison of outcome variables before treatment with adalimumab and at the final visit.

Variable	Week 0 (baseline)	Month 6 (final visit)	P value
Anterior chamber inflammation	2.02 (1.16)	0.42 (0.62)	<0.001
Posterior chamber inflammation	2.38 (2.97)	0.35 (0.71)	<0.005
Macular thickness, *μ*	304.54 (125.03)	230.87 (31.12)	<0.001
Immunosuppression load	8.10 (3.99)	5.08 (3.76)	<0.001

Data as mean (standard deviation).
